# The Relationship between Amygdala Activation and Passive Exposure Time to an Aversive Cue during a Continuous Performance Task

**DOI:** 10.1371/journal.pone.0015093

**Published:** 2010-11-29

**Authors:** Irina A. Strigo, Alan N. Simmons, Scott C. Matthews, Arthur D. (Bud) Craig

**Affiliations:** 1 University of California San Diego, La Jolla, California, United States of America; 2 Veterans Affairs San Diego Healthcare System, San Diego, California, United States of America; 3 Division of Neurosurgery, Barrow Neurological Institute, Phoenix, Arizona, United States of America; University of Sheffield, United Kingdom

## Abstract

The allocation of attention modulates negative emotional processing in the amygdala. However, the role of passive exposure time to emotional signals in the modulation of amygdala activity during active task performance has not been examined. In two functional Magnetic Resonance Imaging (fMRI) experiments conducted in two different groups of healthy human subjects, we examined activation in the amygdala due to cued anticipation of painful stimuli while subjects performed a simple continuous performance task (CPT) with either a fixed or a parametrically varied trial duration. In the first experiment (N = 16), engagement in the CPT during a task with fixed trial duration produced the expected attenuation of amygdala activation, but close analysis suggested that the attenuation occurred during the period of active engagement in CPT, and that amygdala activity increased proportionately during the remainder of each trial, when subjects were passively exposed to the pain cue. In the second experiment (N = 12), the duration of each trial was parametrically varied, and we found that amygdala activation was linearly related to the time of passive exposure to the anticipatory cue. We suggest that amygdala activation during negative anticipatory processing depends directly on the passive exposure time to the negative cue.

## Introduction

Seminal experiments in animals have identified the neurobiological basis of the conditioned fear response, i.e., freezing behavior and associated autonomic and endocrine responses in relation to anticipation of a fear-arousing stimulus [1], [2]. The lateral and central nuclei of the amygdala play a pivotal role in this process by activating: (1) the periaqueductal gray matter (PAG) to elicit immobility, (2) the lateral hypothalamus to induce autonomic arousal, and (3) the paraventricular nucleus of the hypothalamus to activate adaptive endocrine responses. This brain network underlies the so-called “passive fear reactions”. These behaviors resemble the cluster of symptoms observed in psychiatric disorders, such as major depressive disorder (MDD), which are characterized by “passive coping” (helplessness) and alterations in autonomic and endocrine functioning [3]. It is plausible that during passive exposure to a terrifying stimulus the available attentional resources are directed toward the fear-arousing stimulus. Recent evidence in animals indicates that passive fear responses can be reduced if the animal engages in a motor action during the occurrence of the conditioned stimulus [4], [5]. Passive waiting is thus offset by “active coping” [3], or an active engagement in a secondary activity. Due to the brain's finite attentional capacity [6], stimuli that occur simultaneously compete for attentional resources [7]. Therefore during active coping some attentional resources are diverted away from the fear-arousing stimulus to the active motor task.

Translational human research suggests that a similar brain mechanism is engaged in humans if active engagement in a secondary task occurs during the anticipation or experience of emotional stimuli, referred to as active coping. Specifically, diverting attentional resources away from emotional, fear-arousing stimuli with an active task leads to a reduction of emotional responses and brain activation within the amygdala and related circuitry [8], [9], [10], [11], [12], [13], [14], [15], [16]. Prior studies have compared behavioral and neural reactivity with and without attentional load (e.g., n-back task [8], Stroop interference task [13]) or between different levels of attentional load (e.g., 0- back task, 2-back task) to measure the effects of active coping on emotional response.

In everyday life during a stressful situation “active coping” probably occurs sequentially with passive waiting and these two processes may even compete in time [5]. Consider the example of waiting for a painful medical procedure in the doctor's office. Almost everyone has experienced the discomfort of sitting in the waiting room as time slowly passes. Salient cues in the waiting room (e.g., a nurse walking by, a door to the doctor's office opening) will often arouse fear. One can choose to passively wait for the appointment, or to engage in active coping by reading a magazine, solving a sudoku puzzle or fantasizing about an upcoming vacation. However, even if we do choose active coping, our minds often stubbornly turn attention back to the salient fear-arousing cues, after which we may again mindfully steer attention back to the magazine article or puzzle. Psychological evidence suggests that the amount of attention we devote or the amount of time we spend focusing on an emotional event is proportional to the emotional impact of that event [17].

Nevertheless, the role of exposure time, rather than cognitive distraction, to emotional stimuli during active coping has not been explicitly addressed in prior studies of amygdala activation during fearful anticipation. We performed two separate functional magnetic resonance imaging (fMRI) studies in two sets of healthy human subjects in order to examine activation in the amygdala during cued anticipation of a painful heat stimulus while subjects were engaged in a continuous performance task (CPT) of either a fixed (Study 1) or a parametrically varied (Study 2) duration. This design allowed us to better characterize the effects of active engagement in a CPT on amygdala activity during anticipation of pain and to explicitly examine the role of time in this process.

## Results

### Rationale of Study 1

**Figure 1:**
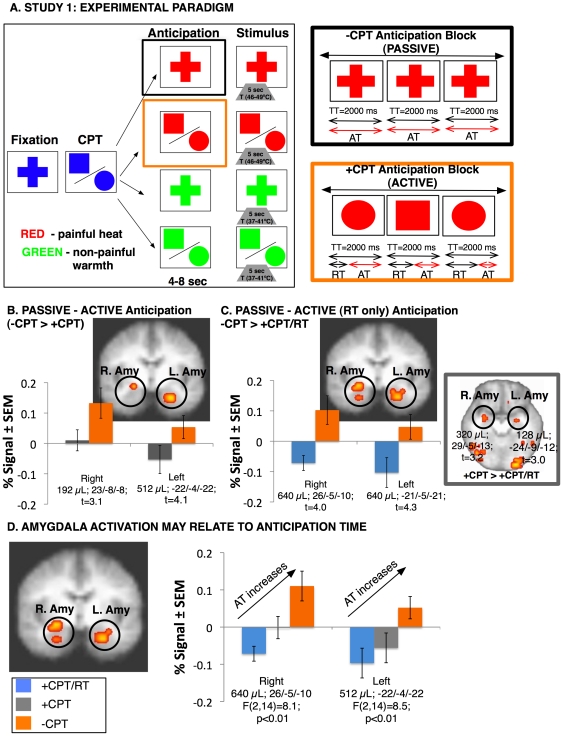
ROI Analyses in Study 1. **A**. A Continuous Performance Task (CPT) was used to induce active coping (circle – LEFT button, square – RIGHT button, fixed rate at 1 trial/2 sec). The stimuli changed color (red – anticipate pain, green – anticipate warmth), 4–8 seconds for the *anticipation condition*. The *stimulus condition* consisted of a hot painful or a warm non-painful stimulus for 5 sec. The two anticipatory conditions of interest are PASSIVE, i.e., −CPT anticipation block and ACTIVE, i.e., +CPT anticipation block. Since the CPT had a fixed trial interval (TT = 2000 msec), each trial can be separated into reaction time period (RT) (i.e., actual engagement) and exposure time to the anticipatory cue of the painful stimulus (AT) (i.e., anticipation time). In this paradigm, RT and AT were always inversely related because the fixed total time (TT) equaled the sum of RT+AT; **B**. Bilateral amygdala activation decreased during CPT consistent with the hypothesis that amygdala activation during aversive anticipation is reduced by engaging in a concomitant task, or by “active coping”. **C**. The decrease in amygdala activation seemed to occur during the time of active engagement in the CPT, i.e., CPT (RT only) period. **D**. Amygdala activation during aversive anticipation may be directly related to the anticipatory time, since it was lowest during RT (AT = 0) and highest during −CPT (AT = TT), with activation during +CPT falling in between (AT = TT-RT).

We conducted functional MRI in 16 healthy subjects while they performed an anticipation paradigm (**Fig. 1A**) used previously by our group [18] (see **METHODS** for details). In the first study we examined the hypothesis that performance of a simple continuous performance task (CPT) during anticipation of painful heat would modulate (i.e., decrease) anticipatory amygdala activity. To test the first hypothesis, we performed ROI analysis within bilateral amygdala and compared amygdala activation during anticipation of painful heat while subjects were engaged in the CPT (+CPT) to activation during anticipation of equally painful heat while subjects were not engaged in the CPT (-CPT) (**Fig. 1A**). We also examined the hypothesis that differential anticipatory amygdala activation between +CPT and –CPT conditions is powered by the actual engagement in the task (measured by the RT period of the +CPT) (**Fig. 1A**). To test this hypothesis, we only modeled the reaction time (RT) period of each +CPT anticipation block in each subject and directly compared amygdala activation between +CPT and +CPT (RT only) periods. This analysis was motivated by the fact that during the RT periods subjects must pay more attention to the task than to the anticipatory cue, whereas during the rest of the +CPT trial subjects can allocate all the time available to the anticipatory cue. Therefore, we expected to have greater amygdala activation during direct comparison of +CPT vs. +CPT (RT only) conditions. Finally, if the anticipation time plays a role in anticipatory amygdala activation, then amygdala activation should be highest during –CPT condition, and lowest during +CPT (RT only) condition with the +CPT condition falling in between. In order to test this hypothesis we performed a repeated measures ANOVA on amygdala activation during the three conditions.

### ROI Analyses – Study 1

#### Decreased bilateral amygdala activation during CPT

As hypothesized, a significant increase in bilateral amygdala activity was observed during anticipation of painfully hot stimulus in the -CPT condition compared to anticipation of equally painful stimulus in the +CPT condition (**Fig. 1B**) (right: 23/-8/-8; t(15) = 3.1; p<0.01; 192 µL; left: -22/-4/-22; t(15) = 4.1; p<0.01; 512 µL). In other words, we observed lower amygdala activation when subjects were actively engaged in the CPT compared to when they passively waited for the same thermal stimuli (**Fig. 1B**). Active engagement in CPT also decreased subjective pain experience in our subjects (see Supporting Information S1).

#### Decreased amygdala activation during +CPT relates to RT period

As hypothesized, RT period, i.e., active engagement in the CPT, powered the attenuation of amygdala activity during +CPT condition. Greater differences in bilateral amygdala activation were observed between passive waiting and active engagement when only RT period was considered (**Fig. 1C**) (right: 26/-5/-10, t(15) = 4.0; p<0.01; 640 µL; left: -21/-5/-21; t(15) = 4.3; p<0.01; 640 µL). Furthermore, amygdala activation was significantly greater during the +CPT relative to +CPT/RT conditions (**Fig. 1C** **- insert**) (right: 29/-5/-13; t(15) = 3.2, p<0.01; 320 µL; left: -24/-9/-12; t(15) = 3.0; p<0.01; 128 µL), suggesting that during the AT period of heat anticipation in the +CPT condition, i.e., during passive exposure to the anticipatory cue following engagement in each CPT trial, amygdala activity increased.

#### Anticipatory amygdala activation seems to be influenced by the anticipatory time

Direct comparison of amygdala activity during the RT period of +CPT to that during +CPT and -CPT conditions showed a linear relationship (**Fig. 1D**), with the lowest amygdala activation during the RT period of +CPT condition and the highest during -CPT condition. This relationship was in direct agreement with the anticipatory time (AT), which was lowest during the RT (AT = 0) period of +CPT condition and highest during the -CPT condition (AT = TT).

### Summary of Study 1 conclusions

1. By comparing active coping (+CPT) to passive waiting (-CPT) we confirmed the hypothesis that amygdala activation during anticipation of painful heat can be effectively reduced by active engagement in a simple task.
2. By separating the reaction time period (RT) from the remaining anticipation period (AT) within +CPT condition we were able to dissect the sequential nature of coping with fear-arousing cues and found that attenuation of amygdala activation seemed to occur during the RT period of the task, i.e., during the time of active engagement in the CPT, and in fact increased during the AT period of the task.
3. By directly comparing amygdala activation during +CPT (RT only), +CPT and -CPT periods we gained initial support for greater amygdala activation as a function of AT.

Therefore, we showed that the exposure time to salient cues, which is highest during -CPT and lowest during the RT period of +CPT, seems to influence amygdala activation during anticipation of painful heat. However, in Study 1, we used a fixed trial duration, thus we could not disambiguate between an effect of anticipation time from an effect of reaction time. This is potentially important if amygdala responses can be explained by neural activity during the reaction time that reflects non-specific or cognitive (e.g., attentional) differences between trials or subjects. In other words, to confirm that active coping did indeed suppress amygdala responses to the anticipation of pain, we needed to show that varying the anticipation time, independently of the reaction time, can cause changes in amygdala responses. To do this we conducted a follow-up study where we parametrically varied the trial duration across three levels and repeated the experiment.

### Rationale of Study 2

**Figure 2:**
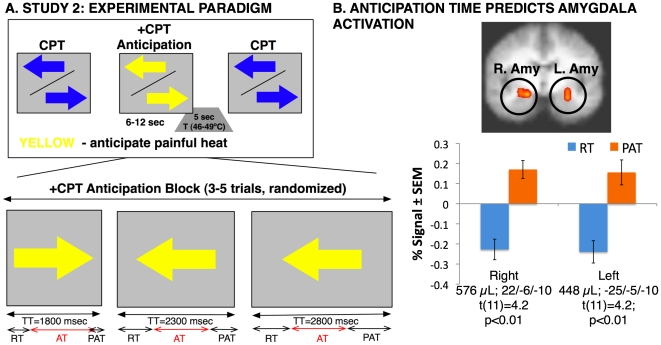
ROI Analyses in Study 2. **A**. A Continuous Performance Task (CPT) was used to induce active coping (left arrow – LEFT button, right arrow – RIGHT button). Total trial duration was modulated parametrically (TT = 1800, 2300, or 2800 msec) in a pseudorandomly balanced manner. The stimuli changed color (blue/yellow (50% of subjects) or yellow/blue (50% subjects) – anticipate pain, 6–12 seconds, 3–5 trials — to signal an impending noxious heat stimulus (*anticipation condition*). The *stimulus condition* consisted of an individualized hot painful stimulus for 5 seconds. **B**. Amygdala activity was positively correlated with the parametrically modulated trial duration (PAT), consistent with the time-dependent model of amygdala activation.

A novel anticipation paradigm (**Fig. 2A**) was administered during fMRI to 12 additional healthy subjects (see Methods). As in Study 1, we first identified for each subject and each trial the (1) RT (reaction time), i.e., the specific time of active engagement in the CPT during the pain anticipation trials. Next, in order to delink the anticipation time (AT) from the RT (in contrast to Study 1), each trial contained an additional (2) PAT (parametric anticipation time). Thus, PAT =  (TT –1800)  =  AT + (0, 500, or 1000) msec, so that the exposure time to a salient pain cue was independent of RT. Therefore, each anticipation block could be modeled as a combination of RT, i.e., behavior-dependent anticipation, and PAT, behavior-independent anticipation, (**Fig. 2A**), which were decoupled in time (in the current sample, the within-trial duration of RT and PAT were not significantly correlated; r∼0.06, p = NS). This design allowed us to test the hypothesis that duration-dependent responses in the amygdala under parametrically varied passive anticipation (PAT) were greater than those during the reaction time (RT), while accounting for non-specific (non-duration dependent) response components, such as sensory-motor processing.

### ROI Analyses – Study 2

#### Amygdala activation during anticipation is related to anticipatory time

Parametric modulation of the exposure time to a salient pain cue resulted in a highly significant increase of activation within bilateral amygdala compared to that during RT (**Fig. 2B**), i.e., amygdala activity increased during PAT in direct proportion to the duration of the PAT (right: 22/-6/-10, t(11) = 4.2, p<0.01; 576 µL; left: -25/-5/-10; t(11) = 4.2; p<0.01; 448 µL). These results support the conclusion that amygdala activation during passive exposure to the salient pain cue was directly proportional to the parametrically modulated exposure time.

### Summary of Study 2 conclusions

1. By separately modeling amygdala activation during RT in a task with parametrically varied exposure time we confirmed that attenuation of amygdala activation occurs during the RT period of the task, i.e., during the time of active engagement in the CPT.
2. By parametrically altering the duration of each trial we obtained strong evidence that exposure time (PAT) itself directly modulates amygdala activation.

## Discussion

In two separate experiments with two different groups of healthy volunteers, we systematically examined the effects of active engagement in a CPT (a model of “active coping”) on amygdala activity during cued anticipation of pain. Our results indicate that active engagement in an attentional task attenuates bilateral amygdala activation during anticipation of pain and the perceived pain experience (see Supporting Information S1). These findings strongly support the idea that active coping is an effective strategy for reducing anticipatory anxiety [3]. Our results corroborate previous research on the role of the amygdala in attentional resource allocation during emotional and cognitive processing [19]. In addition, we show that passive exposure to the anticipatory cue produces amygdala activation during anticipation of pain that is independent from the deactivation induced by active engagement in a cognitive task. The present findings provide the first evidence that attenuation of amygdala activity during pain anticipation occurs only during active engagement in the task. Outside of that engagement, amygdala activation occurs that is proportional to the exposure time to the salient, fear-arousing cue. Thus, our study explicitly delineates the time-dependent behavior of the amygdala response when uncoupled from attention during anticipation of heat pain.

To our knowledge, this is the first report that emotion-related amygdala activation is time-dependent. This time-dependent model can parsimoniously explain prior findings on interactions between emotional and cognitive processing that had previously been interpreted as effects of attentional allocation. For instance, Erk et al. (2006) [8] manipulated anticipation of negative images with two levels of difficulty of a working memory task (0-back, 2-back). They found more amygdala activation during anticipation of negative images in a 0-back than in a 2-back condition. The subjects' reaction times in a 0-back condition were significantly faster that in a 2-back condition, which was interpreted as evidence for an attentional load effect; however, our findings suggest an alternative interpretation, viz. that more time remained available for anticipation. Herwig et al. (2007) [20] asked a group of healthy subjects to use a cognitive “control strategy” during aversive anticipation and compared their brain activity to another group of healthy controls that were passively expecting the same images. They concluded that cognitive control exerted during emotional anticipation inhibits regions involved in emotion processing, such as amygdala. Our findings suggest that it is important to recognize that the latter group allocated the entire time to anticipation and showed increased anticipatory amygdala activation, whereas the former group allocated some of the time to performance of the cognitive task and thus less time to actual anticipation, which resulted in attenuated amygdala activity. Blair et al. (2007) [10] examined the effects of a cognitive task with three difficulty levels (no task, easy task, hard task) on negative emotional processing. Amygdala activity showed a monotonic inverse relationship with task difficulty, with the highest activation observed during “no task” condition and smallest activation observed during “hard task” condition. Once again, subjects' reaction times on these tasks showed an inverse relationship with amygdala activity, consistent with the interpretation that more time devoted to the task left less time devoted to emotional processing and produced less amygdala activity. Schaefer et al. (2002) [21] showed that maintaining a negative emotion, i.e., allocating time to process it after the emotional stimulus, produced increased amygdala activation compared to a non-maintained condition. Studies by Pessoa et al. (2002), Van Reekum (2007), and Schaefer et al. (2006) [9], [22], [23] can similarly be re-interpreted as time-dependent. Finally, Dalton and colleagues (2005) [24] found a strong positive correlation between gaze fixation (focus time) and amygdala activity in autistic individuals, and a similar trend in the control subjects. Our results recommend that the time-dependent model be incorporated along with an attention-dependent model to provide the most complete understanding of these findings by showing directly that amygdala activation during anticipation of an aversive emotional stimulus is predicted parametrically by the exposure time to that stimulus.

Our findings can also potentially explain discrepancies in anticipatory amygdala activation between studies. For example, in two similar studies of cued anticipation, significant anticipatory amygdala activity was observed in one [22] but not in the other [25]. In the former study, subjects passively viewed emotional images, and thus the entire block was allocated to anticipation, whereas in the latter study subjects were actively engaged in a continuous performance task, and thus the time was shared between the task and anticipation.

To our knowledge, this is the first study to address the time-dependent activation within amygdala during anticipation of pain. It is important to note, that even with the fast brain imaging techniques (TR = 1 sec) employed, we are limited in our ability to completely separate events that are close in time, as could be accomplished with MEG or EEG. Future studies using even more rapid imaging or modified methods are needed to test and build upon our model. For example, future studies should examine if continuously engaging subjects in a cognitive activity with no time to spare in between the two successive trials completely abolishes amygdala activation (as our results would propose). In addition, future studies could more dramatically vary the implicit passive anticipatory time (e.g., ∼500–9000 sec) and explicitly model each duration.

The ability to control emotional reactions is necessary for survival in complex social and emotional environments [26]. Modeling time-dependent allocation of amygdala resources can potentially enhance the understanding of prior imaging research, as well as brain-behavior relationships during passive waiting and active coping in clinical populations. Future studies are needed that investigate the role of time-dependent amygdala activation in other settings in order to create a unified generalizable model.

## Methods

Ethics Statement: All subjects provided written informed consent, which was approved by the University of California San Diego Human Research Protection Program.

### Study 1

#### Subjects

Seventeen healthy subjects (7 M) age 23.7 years (range 19–37) with an average of 13.4 years of education (range 13–15) participated in this study, which was approved by the University of California San Diego Human Research Protection Program. Each subject completed the structured clinical interview for DSM-IV (SCID-P) [27]. Subjects were excluded if they met DSM-IV criteria for lifetime alcohol or substance dependence; alcohol or substance abuse within the past 30 days; lifetime mood, anxiety, psychotic or other Axis I disorder; had a clinically significant comorbid medical condition (i.e., cardiovascular and/or neurological abnormality); had a history of an acute or chronic pain condition; or had an implanted or non-removable ferromagnetic object.

#### Task Design

An anticipation paradigm (**Fig. 1A**) used previously by our group [18] was administered during functional magnetic resonance imaging (fMRI). This paradigm combined intermittent engagement in a continuous performance task (CPT) with the cued occurrence of thermal stimuli and had two temporal phases (i.e., anticipation and stimulation), two levels of attentional load (i.e., +CPT and -CPT) and two levels of stimulus strength (i.e., painful heat and non-painful warmth). Individualized temperatures were used for each participant so that moderately painful (5 sec; 46–49°C) and non-painful (5 sec; 37–41°C) thermal stimuli were delivered in pseudo-random order by a 9 cm^2^ thermode (Medoc TSA-II, Ramat-Yishai, Israel), which was securely attached to subjects' left volar forearm.

During the +CPT condition, subjects were asked to press the LEFT button whenever they saw a circle, and the RIGHT button whenever they saw a square. Visual stimuli were presented at a fixed rate of 0.5 Hz. Reaction time (RT) and percent correct data were collected during the scan. (Data from one female subject could not be recorded, thus results are shown in 16 healthy volunteers.) During the -CPT condition, fixation crosses were presented on the screen and no button presses were required. Subjects were instructed that they would experience a painful stimulus after the color of the shape changed to RED and a non-painful warm stimulus after the color of the shape changed to GREEN. Subjects were explicitly informed about each anticipation condition.

Subjects received a total of 20 painful (10 during +CPT and 10 during -CPT) and 20 non-painful stimuli (10 during +CPT and 10 during -CPT) randomized across the run. The presentation rate of visual stimuli was fixed, and subjects' RT during the task did not influence the rate of appearance of the shapes on the screen. Therefore, subjects spent a portion of the fixed presentation time (2000 msec) of each +CPT trial engaged in the task but the remaining portion of each +CPT trial attending to the temperature cue (i.e., passively engaged in the emotional process of anticipating the stimulus). The time each subject allocated to performing the CPT was measured by the reaction time (RT) and so the remaining time (2000 msec - RT) was the anticipation time (AT) (**Fig. 1A**). This design allowed us to examine how active task engagement would affect amygdala activation during exposure to the emotionally salient stimulus (i.e., anticipation of pain, in this case).

#### fMRI Protocol

Four fMRI runs sensitive to blood oxygenation level-dependent (BOLD) contrast were collected using a 3.0 Tesla GE scanner (T2* weighted echo planar imaging, TR = 2000 ms, TE = 32 ms, flip angle = 90, FOV = 24 cm, 64×64 matrix, 30 2.6-mm 1.4-mm gap axial slices, 238 scans) while subjects performed the above paradigm. FMRI acquisitions were time-locked to the onset of the task. A high-resolution T1-weighted image (FSPGR, TR = 8 ms, TE = 3 ms, TI = 450 ms, flip angle = 12, FOV = 25 cm, 256×256 matrix, 172 sagital slices, 1×0.97×0.97 mm^3^ voxels) was obtained for anatomical reference.

#### fMRI Deconvolution Analysis

Data were analyzed with the Analysis of Functional NeuroImages (AFNI) software [28]. We modeled our BOLD responses with two conventional linear de-convolution models, comprising stimulus functions convolved with a hemodynamic response function. These stimulus functions encoded the trial-specific activations, which we modeled according to our factorial (or parametric) design. In the first model, the following regressors were used: 1) +CPT heat anticipation, i.e., anticipation of painful heat while subjects were engaged in the CPT; 2) −CPT heat anticipation; 3) +CPT warmth anticipation; 4) −CPT warmth anticipation; 5) +CPT heat stimulus; 6) −CPT heat stimulus; 7) +CPT warm stimulus; 8) −CPT warm stimulus. In the second model, regressors (1) and (3) were scaled (parametrically modulated) where the scaling was the reaction time (RT). This design enabled us to test for the main effect of active engagement (+CPT) relative to passive waiting (−CPT) and the effect of actual active engagement (+CTP/RT) relative to passive waiting (−CPT) and, critically, the interaction between the two. We hoped to show that amygdala responses depended upon anticipation time, which was lowest during +CPT/RT and highest during −CPT. Seven nuisance regressors were included: two cue regressors (i.e., warning subjects of the upcoming +CPT/−CPT conditions), one outlier regressor to control for physiological and scanner noise, three movement regressors to account for residual motion (in the roll, pitch, and yaw direction), and regressors for baseline and linear trends to account for signal drifts. A Gaussian filter with full width-half maximum of 4 mm was applied to the voxel-wise percent signal change data to account for individual variation of the anatomical landmarks. Data from each subject were normalized to Talairach coordinates [29]. Since the amygdala was defined a priori, we performed region of interest (ROI) analyses using Talairach-defined bilateral amygdala masks [29]. A threshold adjustment method based on Monte-Carlo simulations as implemented in AFNI function Alphasim was used to guard against identifying false positive areas of activation [30]. Due to small volume correction, a cluster of at least 128 µL in amygdala during the ROI analysis was considered significant. The percent signal within amygdala that survived the threshold/cluster method described above was extracted and compared using planned contrasts: 1) anticipation of heat −CPT versus anticipation of heat +CPT, i.e., to directly examine the degree to which engagement in the CPT affected amygdala activity during aversive anticipation; 2) anticipation of heat +CPT/RT versus anticipation of heat −CPT, i.e., to examine amygdala activation during the period of actual engagement in the CPT; 3) anticipation of heat +CPT versus anticipation of heat +CPT/RT, i.e., to examine the degree to which the period of actual engagement versus the entire engagement period affects amygdala activity, and 4) 2-way ANOVA model with repeated measures (3dANOVA2) with anticipation (fixed factor: -CPT, +CPT and +CPT/RT) and subject as a random factor to examine whether amygdala activation is proportional to anticipatory time (AT).

### Study 2

#### Subjects

Twelve healthy subjects (5 M) age 20.5 years (range 19–29) with an average of 14.8 years of education (range 14–20) who satisfied inclusion/exclusion criteria of Study 1 (described above), but did not participate in Study 1, participated in Study 2.

#### Task Design

A novel anticipation paradigm (**Fig. 2**) was administered during functional magnetic resonance imaging (fMRI). This paradigm also combined a continuous performance task (+CPT) with painful temperature stimuli and had two temporal phases (i.e., anticipation and stimulation). Subjects were engaged in the CPT throughout the entire duration of the paradigm and a single temperature level that was subjectively rated as moderately painful (5 sec; 46–49°C) was delivered to subjects' left volar forearm following a cue.

During the +CPT condition, subjects were asked to press the LEFT button whenever they saw an arrow pointing to the left, and the RIGHT button whenever they saw an arrow pointing to the right. Reaction time (RT) data and percent correct were collected during the scan. Subjects were instructed that they would experience a painful stimulus after the color of the arrow changed from blue to yellow (50% of subjects), or from yellow to blue (50% of subjects). Subjects again were explicitly informed about each anticipation condition.

In order to assess whether the exposure time to a salient anticipatory cue that predicts a painful stimulus during active engagement in an attentional task drives amygdala activation irrespective of the degree of effortful engagement (i.e., RT), we parametrically modulated the duration of each trial (i.e., arrow). Trials with three different total durations (total time, TT) were balanced across the entire run (TT = 1800, 2300, or 2800 msec). Therefore, the anticipatory time was divided into a reaction time-dependent anticipation period (AT, like in Study 1), with a value of 1800 msec minus RT, and a reaction time-independent anticipation period, i.e., parametric AT (PAT), with values of 0, 500, and 1000 msec. By decoupling exposure time from reaction time, we examined the unique contributions of these periods to the activity of the amygdala. Subjects received a total of 16 moderately painful stimulations, which resulted in 16 total anticipation periods. The duration of each anticipation period was jittered through parametric modulation of the presentation rate and varied from 6–12 sec or 3–5 arrow trials.

#### fMRI Protocol

Two fMRI runs sensitive to blood oxygenation level-dependent (BOLD) contrast were collected using a 3.0 Tesla GE scanner (T2* weighted echo planar imaging, TR = 1000 ms, TE = 32 ms, flip angle = 90, FOV = 24 cm, 64×64 matrix, 20 2.6-mm 1.4-mm gap axial slices, 350 scans) while subjects performed the above paradigm. FMRI acquisitions were time-locked to the onset of the task. A high-resolution T1-weighted image (FSPGR, TR = 8 ms, TE = 3 ms, TI = 450 ms, flip angle = 12, FOV = 25 cm, 172 sagittal slices, 1×0.97×0.97 mm^3^ voxels) was obtained for anatomical reference.

#### fMRI Deconvolution Analysis

Data were analyzed with the Analysis of Functional NeuroImages (AFNI) software [28]. A linear de-convolution model included two de-coupled regressors for the anticipation phase: 1) RT, 2) PAT and one for the stimulus phase 3) pain. Seven nuisance regressors were included: an outlier regressor to control for physiological and scanner noise, three movement regressors to account for residual motion (in the roll, pitch, and yaw direction), a regressor modeling individual white matter to control for non-specific signals within the brain and reduce the effect of auto-correlations, and regressors for baseline and linear trends to account for signal drifts. A Gaussian filter with full width-half maximum of 4 mm was applied to the voxel-wise percent signal change data to account for individual variation of the anatomical landmarks. Data from each subject were normalized to Talairach coordinates [29]. Since the amygdala was defined *a priori*, we performed region of interest (ROI) analyses using Talairach-defined bilateral amygdala masks [29] (AFNI program 3dROIdraw). In order to directly compare amygdala activation during different anticipatory periods we performed planned paired t-test between RT and PAT. A threshold adjustment method based on Monte-Carlo simulations (AFNI program Alphasim) was used to guard against identifying false positive areas of activation similar to Study 1 [30].

## Supporting Information

Supporting Information S1 (DOC)
