# Supplementary material for: The Relationship between Amygdala Activation and Passive Exposure Time to an Aversive Cue during a Continuous Performance Task
Source: PLoS One. 2010 Nov 29;5(11):e15093. doi: 10.1371/journal.pone.0015093 (PMC2993966; doi:10.1371/journal.pone.0015093)

In order to examine whether “active coping” by engaging in a simple motor activity, such as continuous performance task (CPT) modulates (i.e., decreases) subjective pain experience, each subject in Study 1 completed a modified, shorter version of the task one week prior to scanning. In this version, subjects were asked to rate the perceived intensity and unpleasantness of each temperature stimulation immediately following its application (0 – no pain/unpleasantness to 10 – extreme pain/unpleasantness). This allowed us to compare the perceived pain experience (both intensity and unpleasantness) of the identical painful temperature stimulus with (+CPT) and without (-CPT) active coping by engagement in CPT. As expected, both the perceived pain intensity (t(15)=3.6, p<0.01) and pain unpleasantness (t(15)= 2.6, p<0.05) were significantly lower in the +CPT compared to –CPT condition, suggesting that **a simple motor action was sufficient to decrease subjective pain experience.**

**
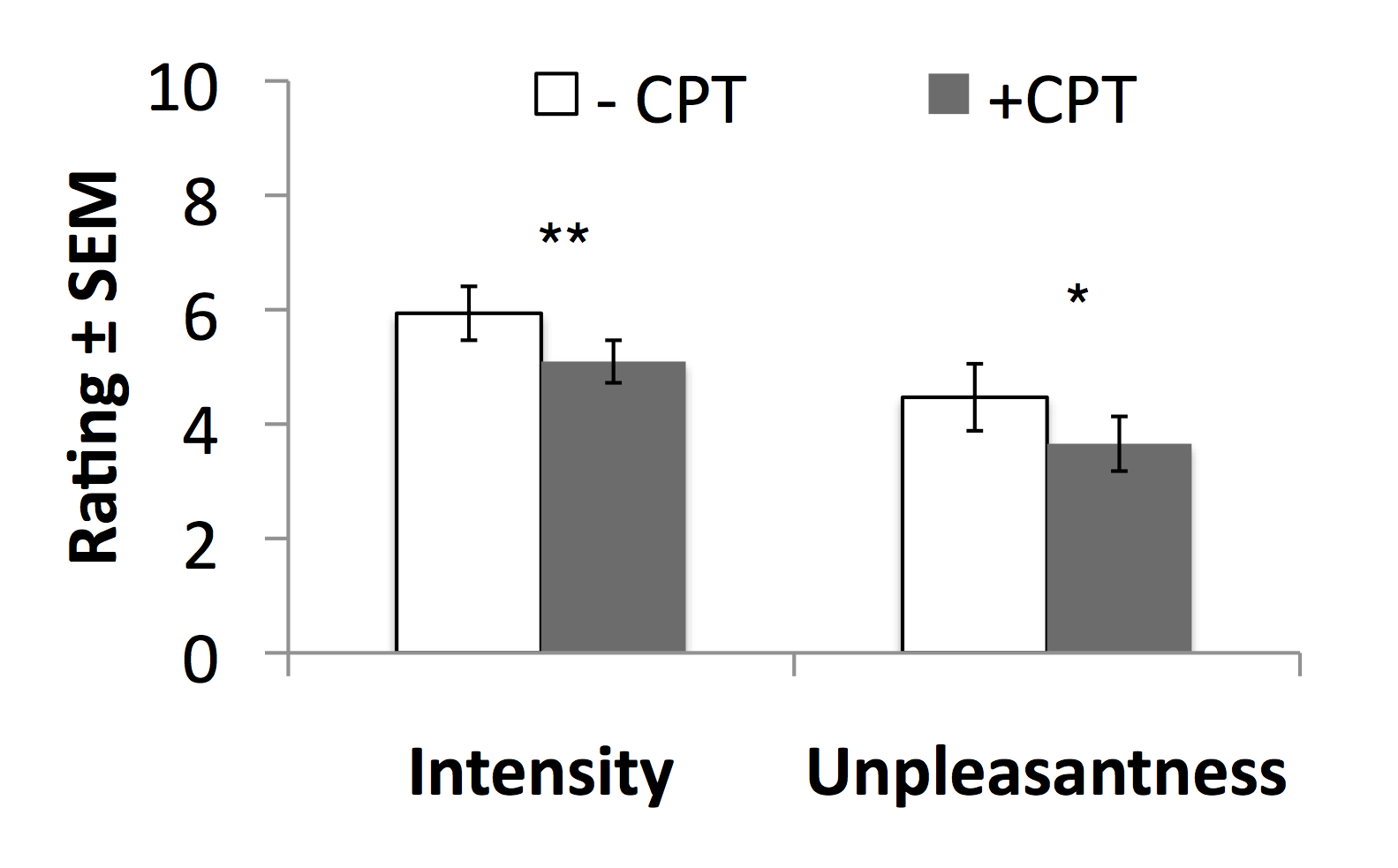
**

We also explored, whether the degree of active coping (measured by subjects RT or engagement in CPT) during anticipation of pain, related to perceived pain experience. We thus correlated individual subjects RT during anticipation of pain with corresponding pain ratings, i.e., perceived pain experience in the +CPT condition. We found significant positive relationship between subjects’ RT and their perceived unpleasantness scores (r=0.5, p<0.05). This indicates that subjects with the shortest RT or fastest responses during anticipation of pain or the highest degree of active coping, also had the lowest perceived pain unpleasantness. Similar, although non-significant relationship was observed with the perceived pain intensity ratings (r=0.3, p=0.2).


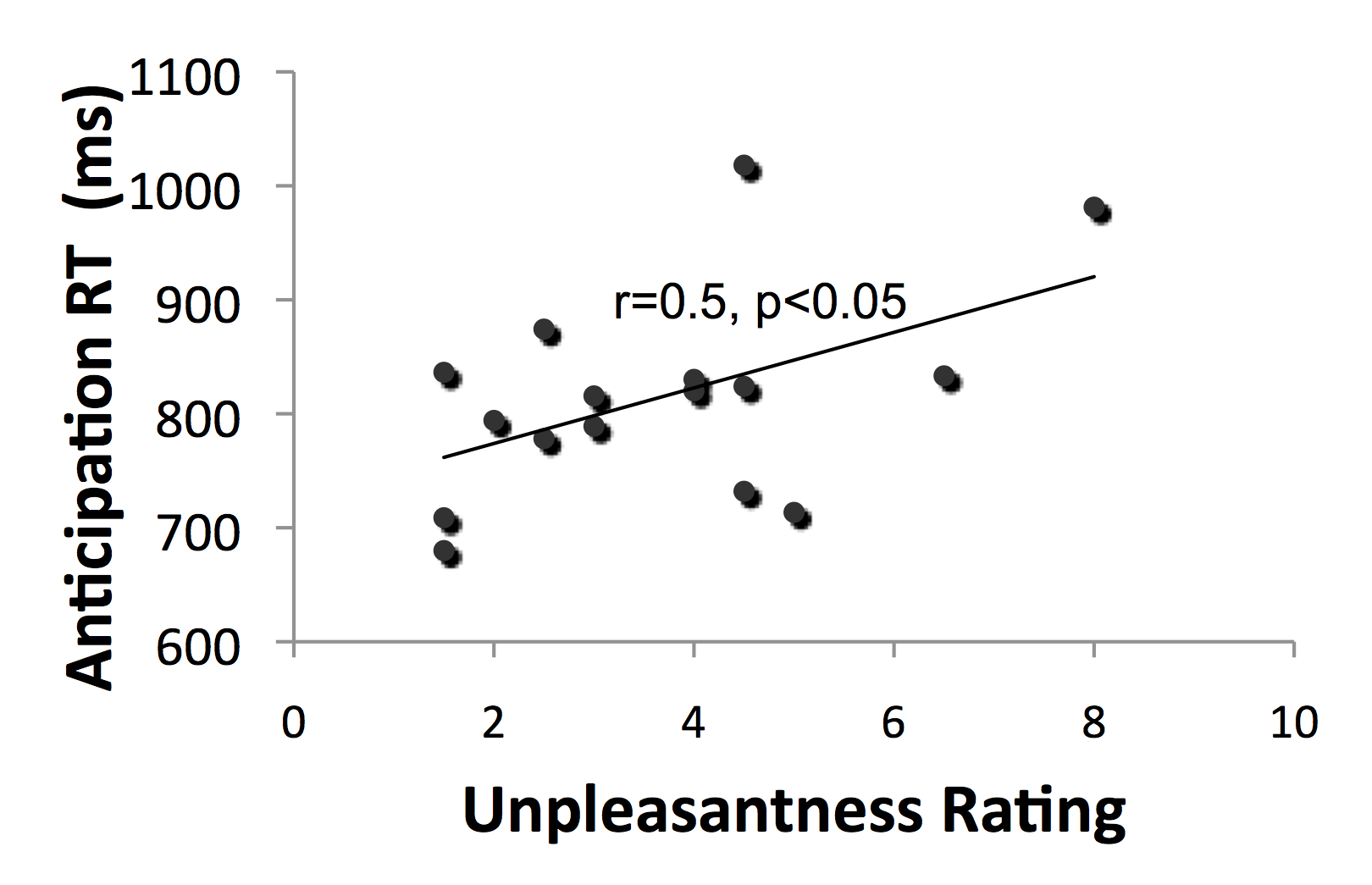

Supplement: Supporting Information S1 — (DOC) [file pone.0015093.s001.doc]
